# How to Successfully Develop Treatments for Early-Stage CKD: A Patient Perspective

**DOI:** 10.1016/j.xkme.2022.100441

**Published:** 2022-03-08

**Authors:** Robert Friedman

**Affiliations:** National Kidney Foundation, New York, NY

Related article, p ●

Successful early-stage treatment of chronic kidney disease (CKD) demands an understanding of how we see ourselves in relation to kidney disease. Professionals must build awareness and create options; however, therapeutic choices will only be exercised when there exists an appreciation on the part of patient and practitioner alike, of what it means to live with kidney disease.

In December 2020, the National Kidney Foundation and the US Food and Drug Administration cosponsored a scientific workshop, highlighting that treatment of CKD in its earlier stages could yield long-term benefits.^1^ Although workshop participants recognized the need for public health campaigns and patient education, we need more than just marketing to change the current paradigm that focuses on treating later-stage CKD. We need to rewrite “the kidney story” in the public and professional imagination.

When I was diagnosed with kidney failure after 30 years of managing type 1 diabetes, I knew little about this disease and even less about what this diagnosis might portend. My doctor did little to ease my journey. In the ensuing decade, as I transitioned from diagnosis to dialysis and then transplant, my family rode a roller coaster of near-death episodes and frustrations. Fortunately, privilege, support, excellent medical care, and my own resilience enabled me to reconnect with the skill set and persona that had once characterized my professional self, and I became an activist in the kidney community. Along the way, I was privileged to share in the personal stories of other patients and came to recognize that people were much more than the “dumbed down” version of their medical histories.

Although physicians look at treatment benefits and risks in terms of biomarkers, safety, and outcomes, the calculus for patients is far more complex. Kidney disease is not only a systemic disease and metabolic disrupter but also a devastating life disrupter, intruding our capacity to control our bodies and to retain our identity, hopes, and aspirations.

What does it mean to be unable to work, to care for and support others; to have considered yourself a person of value and status lined up to reap the rewards from a lifetime of sacrifice, only to have that subsumed by this systemic disease? What does it mean to face the diagnosis “end-stage kidney disease,” whose very terminology implies a death sentence? With kidney disease, the distance from former self, life, and relationships increases every day as intimacy fades, joys and pleasures are gone, and the person is reduced to a coded patient bracelet.

We see prison dramas on television, but the half million Americans receiving dialysis are our invisible incarcerated. Professionals see us as depressed, deficient, and defeated; however, there is a far more tragic dimension to the disease. Even late-stage patients still manage to remain caring, social, resilient, and generous. Their mental health needs are ignored as physicians focus on their quantifiable medical issues and ignore the possibility that there even exists a psychopathology stemming from real physiologic changes that affect the patient's mental status. This story of kidney failure that needs to be told is a poignant and tragic story of people in purgatory, but this tale of “learned helplessness” need not be the only “kidney story.” I have heard different “kidney stories” and successful outcomes in terms of quality of life and clinical results, achieved through patients, caregivers, and providers accessing available options to avoid becoming bystanders in their own version of “It’s a Horrible Life.” Even when disease forces us to confront mortality, the ability to feel a sense of control over its course changes the calculus of decision making.

Our diverse patient community, in clinics, waiting rooms, and “advocacy circles,” is filled with persons at all stages of CKD who display immense courage. I have seen how knowledge, socialization, and empowerment are able to activate patients, motivating ordinary people to find ways to confront disease, collaborate with professionals, and harness expertise.

Medicine has left too many patients tossed between doctors and test results. The therapeutic relationship that once was “hands on” is often reduced to blood draws, scans, and data reports. Case histories omit patients’ life histories, relationships, occupations, and environment: doctors fail to develop a holistic view of kidney disease, are less able to develop rapport, may miss many subclinical cues, and lack insight into the motivators that might bring patients into a treatment plan consistent with their culture, values and lifestyle.

So how to engage physicians and patients when a disease is often undiagnosed, its risks uncertain, and its symptoms not yet manifest? Many other “diseases” have succeeded in engaging the public before their symptoms present. From cigarette smoking to high cholesterol, breast cancer, and prostate cancer, awareness and attitudinal change have led to enormous behavioral change. All clinical treatments rely on cultural change to transition from efficacy to effectiveness, and this is especially true of early-stage CKD prevention.

Drug development to treat people with earlier-stage CKD requires a high safety profile and mechanisms for evaluation of long-term side effects and follow-up. To the extent that the “new wave” of treatments will yield positive benefits related to diabetes, cardiovascular disease, and overall mortality, the case for their use and acceptance becomes ever more convincing. But beyond this, the lack of research in psychologic, attitudinal, and behavioral areas affecting the kidney patient community stymies our connecting medicine with people. Medical anthropology, which studies health and health care in the context of an ecological and community perspective, can potentially yield insight into how to address this challenge. Recent studies using such research are guiding efforts to bring health care to heretofore “difficult to serve” homeless populations and can change practice, culture, and outcomes. Sorely lacking, and much needed, is a medical anthropology of kidney disease.

The workshop paid scant attention to relevant socioeconomic factors. Insofar as there are economic incentives to treat diseases rather than to prevent them, a greater issue in early CKD prevention is not just treatment but rather how and why to address and mitigate known “risk factors” that create a “sick society.” Public health in America, today, is defined by the business of health care and rarely asks how broader interventions might produce fundamental individual and societal benefits. Traditional cost-benefit analysis rarely measures the return on social investment in terms of long-term gains in health outcomes and worker productivity. The narrowness of our approach is such that we continue to advise financially disadvantaged patients with kidney diseases to eat well, while remaining oblivious to cutbacks in the Federal Food Stamps budget.

### Developing Common Ground

It is not enough to aspire to equity in a system that too often fails broad populations, as seen in plummeting health indicators, even before the coronavirus disease 2019 pandemic. The lack of access to the highest standard of preventive, primary, and specialized care; lack of uniformity in costs of care; and inability to use data for accountability, research, planning, and clinical decision making all lessen the potential of America’s massive investment in health care. Although many of these areas are being reckoned with by patients, professionals, and policymakers, existing gaps reflect real philosophic differences as well as economic and political realities. If stakeholders present competing and conflicting perspectives on priorities and the definition of value (Table 1), chances of societal success are diminished.

**Table 1:** Stakeholder Perspectives and Focus

Stakeholder	Priority
Clinicians	Epidemiology, safety, and outcomes related to target conditions and populations; care of the patient in front of them
Payers (including government)	Expenditures, particularly over a shorter time horizon, distributed over a population of beneficiaries
Industry	Profitability, risk to reward ratio, and market share and forecasts, focused more on the short term
Patients	Alleviation of symptoms and disease, condition maintenance, and/or improvement in quality of life

These priorities represent both tangible differences and ingrained ways of thinking. To reconcile these perspectives within the current health care system, policymakers must resist corrupting influences, reckon with these differences, and openly seek out and incentivize common ground. We need to move beyond the ledger sheets to develop a shared interest in achieving such broader economic and social goals. Investment in health care is predicated upon the concept of “return on investment”; however, American economists have too often looked only at the direct costs of disease and made little effort to quantify beyond that. We need to understand how kidney disease takes a toll far beyond direct expense and how investment in prevention and treatment can “pay off.” Reform must evaluate how to reconfigure “all” institutions, policies, and practices. This will often stray from medicine’s traditional inclination to be nonpartisan and apolitical, but insofar as physical, mental, economic, and social health are intrinsically linked, medicine cannot stay within a narrow line, any more than a nephrologist can look at kidneys apart from the whole of the human body.

### Moving Toward a More Coherent System

A highly effective coalescing of patient and physician organizations, communities, the government, and health care industries can be seen in the 2019 Advancing American Kidney Health Executive Order. The Kidney Precision Medicine Project, the activities of the Patient-Centered Outcomes Research Institute, and the Kidney X Innovation Incubator each reflect meaningful patient input. Comprehensive chronic disease care programs, such as through the Center for Medicare and Medicaid Innovation, from a single point of comprehensive service to interorganizational networks, target a seamless continuum of medical and supportive services, often with superior outcomes.

Better relationships among primary care practitioners, endocrinologists, cardiologists, and nephrologists are already being targeted by patient and professional groups, such as the National Kidney Foundation, the American Society of Nephrology, the American Association of Kidney Patients, the American Diabetes Association, and the American Heart Association, and can be achieved through education and collaboration; through aligned design of diagnostic and treatment protocols; and through the use of interoperable definitions, terminology, and patient records. But connections need to also extend to constituencies on the grassroots level, engaging community leaders, nongovernmental organizations, faith-based entities, and other “social influencers” to help craft a common language for the “kidney story” and connect with trial participants, families, and patients.

From centuries ago, when the human kidney was just an organ on an anatomy chart, to the modern era when medicine has developed interventions to slow or alter the course of kidney disease, the science around much of what the kidney does and why kidney disease occurs remains elusive. At the same time, the “kidney community” has shown a remarkable ability to develop practical solutions that can rewrite the “kidney story,” and through prevention, early-stage treatment, and significant advances in what was formerly considered “end-stage” kidney disease, to highlight how medicine can respond to human needs and can write a “happy ending” to the stories of patients.
